# Peripapillary hyper-reflective ovoid mass-like structures (PHOMS): clinical significance, associations, and prognostic implications in ophthalmic conditions

**DOI:** 10.3389/fneur.2023.1190279

**Published:** 2023-05-18

**Authors:** Biao Li, Haoran Li, Qun Huang, Yanlin Zheng

**Affiliations:** Chengdu University of Traditional Chinese Medicine, Chengdu, Sichuan, China

**Keywords:** peripapillary hyper-reflective ovoid mass-like structures, optic disc drusen, papilledema, myopic/tilted optic discs, optic neuritis, multiple sclerosis, NA-AION

## Abstract

Pioneering advancements in optical coherence tomography (OCT) have facilitated the discernment of peripapillary hyper-reflective ovoid mass-like structures (PHOMS), prevalent neuro-ophthalmological findings associated with an array of ophthalmic conditions, such as optic disc drusen (ODD), papilledema, myopic/tilted optic discs, non-arteritic anterior ischemic optic neuropathy (NA-AION), and optic neuritis. Despite an expanding corpus of research, numerous inquiries persist concerning their clinical significance, correlations with ocular afflictions, and prognostic implications. This comprehensive review endeavors to impart an in-depth comprehension of PHOMS, encompassing facets like conceptualization, detection, pathogenesis, and associations with diverse ophthalmic conditions. Furthermore, we underscore several unresolved quandaries and suggest prospective avenues for future exploration.

## 1. Introduction

The advent of OCT has precipitated a paradigm shift in retinal and optic nerve imaging. With the introduction of spectral-domain OCT (SD-OCT), visualization of the peripapillary region and the deep prelaminar optic nerve head has been significantly enhanced (1). Enhanced Depth Imaging OCT (EDI-OCT) has markedly improved both imaging speed and depth penetration, enabling more precise and straightforward *in vivo* observation of optic disc anatomical structures (2). The widespread adoption of SD-OCT and EDI-OCT has led to the identification of peripapillary hyper-reflective ovoid mass-like structures (PHOMS) as a common neuro-ophthalmological OCT finding.

PHOMS frequently manifest in OCT imaging of the optic disc and are presently believed to originate from localized distortion and folding of optic nerve fiber bundles as they exit the lamina cribrosa and extend toward the surrounding retina (3). PHOMS have been discerned in a multitude of ophthalmic conditions, encompassing ODD, papilledema, myopic/tilted optic discs, NA-AION, and optic neuritis (2, 4–10).

Illuminating the associations between these conditions and PHOMS can expedite precise identification and intervention. In this comprehensive review, we endeavor to impart an in-depth comprehension of PHOMS, embracing facets such as conceptualization, identification, pathogenesis, and associations with diverse ophthalmic conditions. Additionally, we accentuate numerous unresolved conundrums concerning PHOMS and suggest potential directions for forthcoming exploration.

## 2. Manuscript formatting

### 2.1. The concept of PHOMS

Currently recognized as PHOMS, these structures were initially misidentified as 'buried optic disc drusen' in 2011 OCT studies. (11). Following this, a succession of investigations into buried optic disc drusen adopted the criteria specific to PHOMS. With advancements in OCT resolution and penetration depth, it became feasible to observe the petal-like morphology of ODD at the optic disc center and boot-shaped structures around the disc periphery using SD-OCT (12). This early form of PHOMS was already visible, yet due to resolution limitations, it remained underappreciated within the ophthalmological community. In 2018, the Optic Disc Drusen Studies (ODDS) Consortium employed EDI-OCT to delineate and introduce the acronym PHOMS, classifying them as distinct from ODD (2).

The appellation of PHOMS originates from the morphological delineation of horizontal B-scans (cross-sectional) of the optic disc, ascertained through EDI-OCT imaging. Characteristically hyper-reflective and ovoid in form, PHOMS displace retinal strata both upward and laterally. Situated external to the optic disc and superior to Bruch's membrane opening (BMO), these entities exhibit a homogeneous, elevated reflectivity signal internally, akin to that of the retinal nerve fiber layer and ganglion cell stratum (2, 13).

The acronym “PHOMS” encapsulates the key features of the entity:

P: Situated in the peripapillary area encircling the optic nerve, specifically at the brink of the Bruch's membrane opening (BMO), PHOMS can expand circumferentially past the BMO boundary and assume a full or partial toroidal arrangement. This correlates ophthalmoscopically with an obscure, pale C-shaped peripapillary “halo” that veils the optic disc margin (4, 9, 14).

H: Exhibiting diffuse hyper-reflectivity, PHOMS indicate inhomogeneous structures with a heterogeneous mixture of substructures visible through histopathologic examination (9, 14, 15).

O: Displaying an ovoid shape in a typical B-scan OCT image, PHOMS possesses a round, cylindrical shape as a partial torus in 3 dimensions (16).

MS: Representing a “Mass-like Structure” due to the three-dimensional structure of PHOMS, capable of displacing two or more overlying retinal strata anteriorly and radially outward (2, 13, 17). This leads to a unique presentation characterized as akin to a “ski slope” (2, 13), “boot shape (11),” or “bend” (4).

Although frequently observed in idiopathic intracranial hypertension (IIH), PHOMS is a non-specific OCT finding present in various other conditions, such as multiple sclerosis(MS)-related optic neuritis, NA-AION, tilted disc syndrome (TDS), and ODD. The incidence of PHOMS varies among these diseases, with occurrence rates reported as approximately 62% in IIHS, 47% in ODD, 44% in anomalous optic discs, 19% in optic neuritis, and 12% in optic atrophy (10, 13). Based on the underlying diseases, PHOMS can be broadly categorized into three types: disk edema-associated PHOMS, ODD-associated PHOMS, or anomalous disk-associated PHOMS (18).

### 2.2. The identification of PHOMS

#### 2.2.1. Features of PHOMS

**Figure 1:**
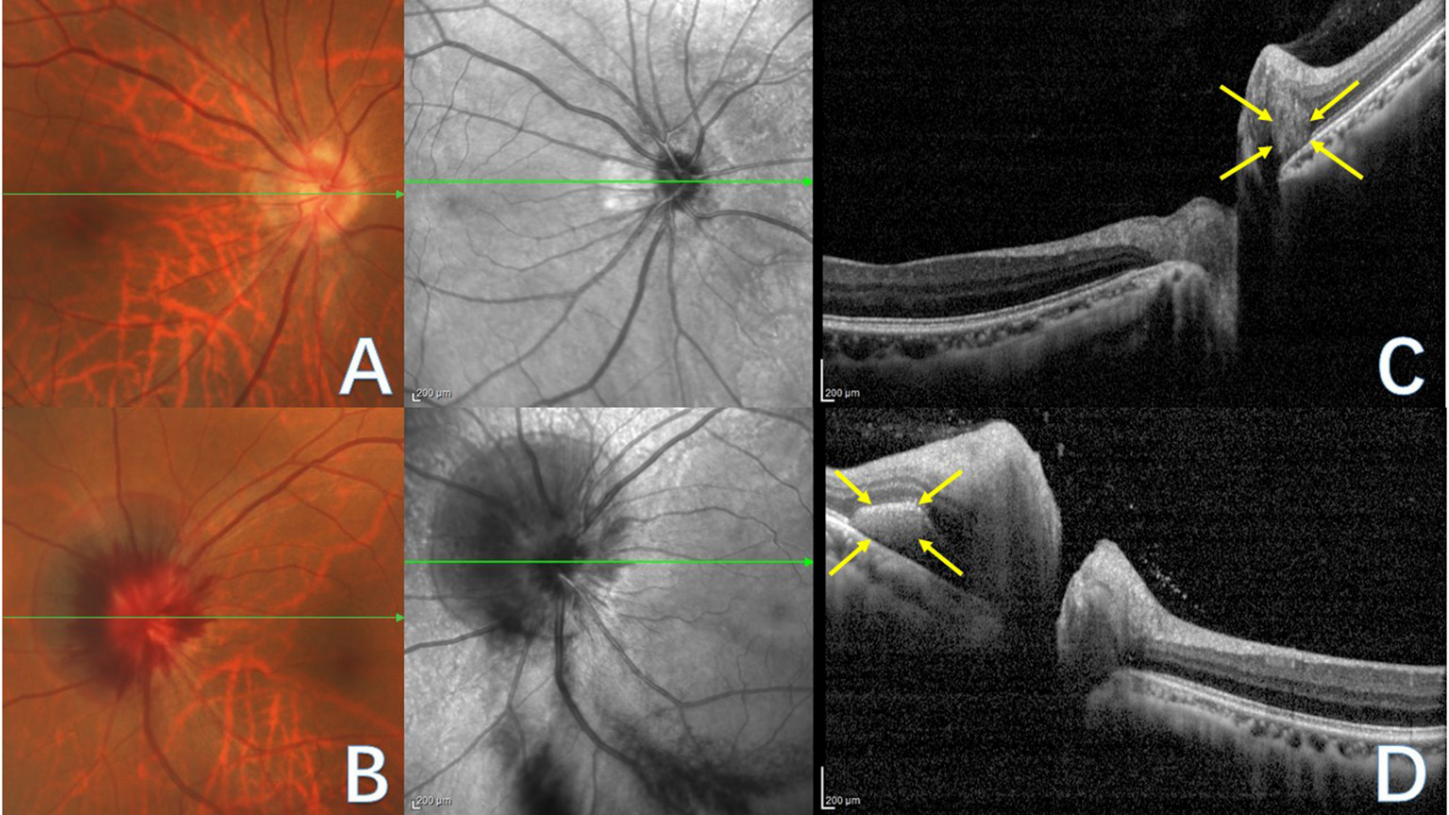
Fundus photograph **(A, B)** and corresponding OCT images **(C, D)** of PHOMS in a patient with tilted optic disc. The green line indicates the OCT scan position. **(A)** Right eye fundus photography reveals blurred optic disc boundaries, nasal tilting of the optic disc, a peripapillary atrophic crescent, and a “C”-shaped halo surrounding the optic disc. **(B)** Left eye fundus photography displays blurred optic disc boundaries and bleeding of the optic disc. **(C)** OCT image of the right eye illustrates PHOMS (yellow arrow), characterized by a hyperreflective ovoid mass-like structure encircled by a hyporeflective edge around the optic disc. **(D)** OCT image of the left eye depicts PHOMS (yellow arrow) in the peripapillary region as a hyperreflective structure that deflects retinal layers upward and laterally; although the left portion of PHOMS appears ovoid, the right portion is obscured by an overlying shadow.

In 2018, the ODDS Consortium characterized PHOMS and advocated EDI-OCT as the benchmark for diagnostic assessment (2). EDI-OCT offers precise visualization of PHOMS' morphological characteristics (16), which are situated outside the optic disc, above the Bruch's membrane opening (BMO), and display a uniform high reflectivity signal internally, akin to the retinal nerve fiber layer and ganglion cell layer (2, 13). PHOMS possess a three-dimensional structure that can displace two or more overlying retinal layers anteriorly and radially outward. In 2D, PHOMS exhibit a “ski slope-like” (13) or “boot-shaped” (11) appearance on B-scan OCT transverse sections (Figures 1C, D).

In optic disc photographs and confocal scanning laser ophthalmoscopic images, PHOMS' ovoid lesions coalesce, forming a circular or C-shaped pattern encircling the optic disc (Figures 1A, B) and appearing as a dome-shaped or doughnut-like structure (19). In 3D, PHOMS may manifest circumferentially around the optic disc, generating a partially torus-like cylindrical shape rather than a discrete mass (2, 16, 20).

Furthermore, high-resolution OCT angiography can identify microvasculature networks within PHOMS of various etiologies (3, 14). For example, Ahn et al. found that vessel density, as measured by OCT angiography, substantially diminished in eyes with large PHOMS but not in those with small or medium PHOMS (21). Fundus examination, another ophthalmological assessment method, can reveal changes in PHOMS morphology, manifested as blurred optic disc margins (7, 13, 15).

Other ophthalmic imaging modalities, such as B-scan ultrasound, computed tomography (CT), or fundus autofluorescence (FAF) imaging, cannot visualize PHOMS (2, 9, 22).

#### 2.2.2. Differential diagnosis of PHOMS

Although PHOMS is highly distinguishable on OCT, its morphology shares similarities with optic disc vasculature and ODD, potentially leading to misdiagnosis.

Optic disc blood vessels exhibit a markedly different appearance on EDI-OCT compared to PHOMS (2, 9, 23). In cross-sectional imaging, blood vessels within the optic disc may display an oval-shaped form with a hyper-reflective interior resembling PHOMS. Nevertheless, they can be distinguished by their sharply defined hyper-reflective anterior and posterior borders, casting elongated shadows upon underlying structures. In longitudinal section imaging, optic disc blood vessels display a long, ribbon-like three-layer profile with strong reflectivity when imaged parallel to the vessel direction. These vessels reside either on the optic disc surface or within it but not adjacent to Bruch's membrane. Conversely, PHOMS are situated in the peripapillary region directly above Bruch's membrane.

Previously considered a form of ODD, PHOMS often coexists with ODD and may resemble ODD on OCT B-scan imaging. Both structures appear ovoid in cross-section and occupy space, with PHOMS approximating the size of a moderate-sized ODD. The primary distinguishing characteristic between these two entities lies in their OCT imaging appearance. Specifically, ODD displays an irregular hyper-reflective rim encircling a hypo-reflective core, while PHOMS presents as a homogeneous mass-like structure without a hypo-reflective core (2, 24, 25). For an in-depth differential diagnosis between ODD and PHOMS, refer to the “The association between ODD and PHOMS” section below.

### 2.3. The pathogenesis of PHOMS

The naming of PHOMS primarily stems from the morphological characteristics observed on cross-sectional imaging of the optic disc using EDI-OCT, while histological studies have mostly focused on the pathology of optic disc edema (ODE) and ODD (26–28).

Histopathological examinations and other evidence suggest that PHOMS may be associated with the lateral protrusion or herniation of swollen axons into the peripapillary retina as they exit the lamina cribrosa and enter the optic disc, extending toward the surrounding retina (3). The formation of PHOMS is associated with axonal swelling and expansion, as well as congenital and acquired abnormalities in optic disc development or the inclination at which the optic nerve penetrates the retina. Hayreh et al. observed that PHOMS is characterized by a bubble-like structure where nerve fibers are dilated, protruding outward, and folding into an S-shape due to severe edema. An ovoid mass-like structure forms at the lower portion of the S-shaped herniation, propelling the peripapillary retinal layers upward, recognized as a salient characteristic of PHOMS (17, 21). The periphery of the nerve fibers is surrounded by a large number of dilated small veins and capillaries. The exudation of interstitial fluid can exacerbate the compression and swelling of nerve fibers in the prelaminar tissue.

The pathophysiological mechanism implicated in PHOMS formation may be axoplasmic stasis. This theory is supported by ophthalmic conditions commonly concomitant with PHOMS, such as ODD, optic disc edema, and TDS (15, 28). Radiolabeled and electron microscopy studies have shown markers of axoplasmic stasis within optic nerve fibers surrounding ODD (9). Adjacent axons to the ODD were found to be compressed and bulging toward the retina on both sides, and this bulging structure presented positive staining for S100 immunostaining, a marker of neural fibers (9, 13, 27). These findings lend credence to the notion that PHOMS arise from axonal transport stasis within nerve tissue. This morphology and location are similar to PHOMS, as seen on OCT imaging. As the optic disc edema subsides, the swelling and vacuolation of the nerve fibers diminish, and the protruding fibers retract into the scleral canal. The markers of stasis axoplasmic vanish, and PHOMS also regresses and disappears.

PHOMS is widely believed to be an indicator of impaired axoplasmic flow, which can result from various factors, including mechanical traction from ODD, papilledema, optic neuritis, and optic disc anomalies, among others (15, 29, 30). Firstly, on pathological sections of ODD, degeneration of nerve fiber cells, intracellular and extracellular calcium deposits, and mechanical compression by dense, rock-like calcification can be observed, and axoplasmic flow stasis of nerve fiber axons also occurs (31). Secondly, studies in primate models of papilledema have shown that the lamina cribrosa's rigid structure compresses the edematous nerve, leading to axoplasmic stasis, as demonstrated by the use of radioactive isotopes (15, 28). Thirdly, in pathological sections of optic neuritis models, Rao found PHOMS, which was accompanied by demyelination of optic nerve fibers, extensive aggregation, and infiltration of inflammatory cells, leading to acute stasis of axoplasmic flow in nerve fibers of the optic disc (32). Fourthly, the nerve fibers of patients with myopia and tilted optic disc undergo severe bending as they enter the lamina cribrosa of the sclera, and the degree of tilt can cause chronic stretching damage to the nerve fibers. This can result in focal stasis of axoplasmic flow, with PHOMS being most commonly observed in the nasal quadrant in cases of optic disc tilt (30).

In summary, various optic nerve diseases, whether acute inflammation of the nerve fibers or chronic mechanical traction damage, lead to the same pathological process of axoplasmic stasis. Microvascular leakage exacerbates nerve fiber damage, ultimately leading to a vicious cycle that causes swelling of the nerve fibers, S-shaped distortion, and displacement, resulting in the formation of oval or nodular PHOMS structures.

It is crucial to understand the different factors that contribute to the formation of PHOMS and the underlying conditions associated with them. This knowledge can help clinicians in making an accurate diagnosis and providing appropriate treatment for patients with ophthalmic diseases related to PHOMS. Further studies on the histopathological characteristics of PHOMS and their associations with various optic nerve diseases are needed to provide a more comprehensive understanding of this phenomenon and to develop more targeted therapeutic strategies for the conditions that cause PHOMS.

### 2.4. The association between PHOMS and other ophthalmic conditions

PHOMS constitute a nonspecific OCT manifestation, not exclusively linked to a single disease entity. Recently, associations between PHOMS and multiple ophthalmic pathologies have been discerned, encompassing but not limited to ODD (2, 5, 9, 10, 33), papilledema (5, 9, 10), myopic or tilted optic discs (4, 7, 9), NA-AION (6, 8–10), and optic neuritis (9, 10). Elucidating the connections between PHOMS and these optic neuropathies facilitates their timely and accurate detection and treatment, highlighting the clinical significance of investigating PHOMS. Moreover, the existence of PHOMS might be associated with disease severity and duration, rendering it a potentially valuable early indicator for various optic neuropathies.

#### 2.4.1. Association between PHOMS and ODD

Originally considered a variant of ODD, PHOMS frequently coexist with ODD and may display analogous cross-sectional OCT B-scan features. ODD are non-cellular calcified deposits within the optic nerve head. The ODDS Consortium characterized ODD's EDI-OCT features as an irregular hyper-reflective rim with a hypo-reflective core, the latter being essential for diagnosing ODD. The Consortium also advised against classifying PHOMS as ODD due to distinct OCT characteristics, B-mode ultrasound, and autofluorescence imaging features (2).

ODD, inorganic calcified deposits frequently observed in the optic nerve head (22, 34, 35), usually remain asymptomatic but may occasionally manifest as acute vision loss, peripheral visual field defects, NA-AION, or pseudopapilledema, among other manifestations (33, 36).

Population-based ODD incidence varies from 1% to 2.4% depending on study methodology (37, 38). Cadaveric research estimated a 2.4% prevalence among the general population (37). Malmqvist et al. discovered ODD in 1% of 1,303 asymptomatic Danish children (38). Pathology examination following enucleation identified ODD prevalence at approximately 1%−2%, with 20% being superficial and 80% buried (5). While once thought to be a form of ODD, PHOMS frequently coexist with ODD. Teixeira et al. observed PHOMS in 90% of pediatric ODD patients (33). Another study demonstrated a 47% ODD and PHOMS association (10). Hamann et al. reviewed 65 NA-AION patients, finding PHOMS in 54% of ODD patients and 28% without ODD. Patients with ODD were statistically more likely to have PHOMS (6).

Both PHOMS and ODD can produce pseudopapilledema by obscuring optic disc margins (21, 24). Although ODD was initially considered the primary pseudopapilledema cause in children, recent research indicates PHOMS as the more frequent contributor. Mezad-Koursh et al.'s (30) study showed that 93.8% of pseudopapilledema cases were PHOMS-associated, with only 20.3% related to ODD (30). Guo et al.'s (39) study on 33 ODD eyes revealed 63.6% exhibiting optic nerve pseudoedema and all with OCT-visible PHOMS (1). Ahn et al.'s (21) study on 45 children (84 eyes) with pseudopapilledema found PHOMS in 53.6% (45 eyes), suggesting PHOMS as a possible cause in children. They also found ODD in 25% of small, 37% of medium, and 50% of large PHOMS eyes (21).

PHOMS may resemble ODD in some aspects, including their imaging morphology, size, and pathological mechanisms. Specifically, on cross-sectional OCT B-scan imaging, a PHOMS can bear a superficial resemblance to a moderate-sized ODD, and both of them appear ovoid and occupy space above the lamina cribrosa (2, 31). PHOMS and ODD have a common anatomical basis of small scleral canals (21) and share a pathological mechanism characterized by nerve fiber edema and stasis of axoplasmic transport (9, 15, 27). The development of PHOMS in ODD may be attributed to the mechanical compression of optic nerve fibers by dense calcified ODD, leading to axoplasmic stasis (15, 27, 31, 40).

Despite similarities in imaging morphology, size, and pathological mechanisms, overwhelming evidence refutes PHOMS as a type of ODD (34). This assertion is based on location, histopathology, appearance, associated conditions, and prognosis.

First, PHOMS primarily occupy peripapillary locations external to and encompassing disc portions (13), whereas ODD typically resides within the optic nerve head, infrequently in the peripapillary region (2).

Second, histopathologically, PHOMS feature optic nerve fiber bulges extending centrifugally across Bruch's membrane opening into peripapillary area (15, 21), interspersed with small dilated capillaries. They also comprise nerve fibers, vacuoles, capillaries, and interstitial fluid compartments (3, 14). In contrast, ODD presents as rounded, acellular hyaline accumulations composed predominantly of transparent crystalline calcium phosphate (22), exhibiting uniform density devoid of substantial internal substructure (23) and lacking vascularization (41).

Third, PHOMS and ODD appearances differ. Using EDI-OCT, PHOMS consistently display an ovoid morphology, demonstrating a homogeneous hyper-reflective signal internally in two dimensions (13) while adopting an entire or partial toroidal structure in three dimensions (16, 19). In contrast, ODD occasionally exhibits a rudimentary oval shape yet frequently reveals a lobulated, intricate configuration or aggregation of ODD clusters characterized by a hyporeflective core and hyperreflective margins. A quintessential solitary ODD manifests as a distinct ellipsoidal “mass” lacking toroidal extension on consecutive OCT slices (2). PHOMS are not detectable with B-scan ultrasound, CT, or FAF imaging, unlike ODD (9, 22, 25). Ophthalmoscopically, PHOMS display a hazy peripapillary halo, most pronounced nasally, while ODD appears as shimmering reflective nodules within the optic disc.

Fourth, conditions typically associated with PHOMS encompass papilledema, ODD, NA-AION, CRVO, optic neuritis, and TDS/myopia, whereas ODD is linked to retinitis pigmentosa, pseudoxanthoma elasticum, Alagille syndrome, and angioid streaks (2).

Lastly, PHOMS prognosis largely depends on the underlying etiology, with the potential for *de novo* development, fluctuation/regression in tandem with the degree of disc edema, or maintaining a static state (9, 10). In contrast, ODD, as acellular calcified deposits, never spontaneously shrink with disease progression and may cause various complications as they slowly grow over decades (42, 43).

#### 2.4.2. Association between PHOMS and IIH/papilledema

PHOMS may induce pseudopapilledema by obscuring the optic disc margins (21, 24). Pseudopapilledema refers to a benign optic disc elevation, mimicking genuine papilledema, which entails optic nerve head edema due to increased intracranial pressure. Unlike true papilledema, pseudopapilledema lacks pathophysiological origins and avoids vision-threatening consequences (43, 44). Papilledema specifically signifies a pathological optic nerve head elevation resulting from elevated intracranial pressure (17), causing optic disc swelling and a potential series of vision-impairing outcomes, including transient visual obscurations, visual field defects, and, severe cases, irreversible vision loss. IIH is an acquired disorder characterized by heightened intracranial pressure, leading to papilledema and axonal injury (28, 45).

The incidence of IIH is estimated at approximately 0.03 to 4.69 per 100,000 individuals, with higher rates observed in overweight individuals and women of childbearing age (28, 45). Recent studies report that the incidence of PHOMS in patients with IIH ranges from 62% to 81.3% (10, 45).

In 2018, Malmqvist et al. documented that, concomitant with the clinical resolution of papilledema and the retraction of centrifugally herniated nerve fibers into the scleral canal, a concurrent regression of PHOMS is frequently observed (9). Wibroe et al. (45) conducted a retrospective analysis on 32 IIH patients (64 eyes) and identified PHOMS in 26 cases (81.3%). No discernible statistical differences were detected between PHOMS regarding follow-up OCT ganglion cell layer thickness, suggesting that PHOMS do not induce pathological damage to the optic disc or macular nerve fibers. The authors did not report any PHOMS disappearance during follow-up, which spanned from 2 months to 7 years, did not mention whether edema had completely subsided at this stage. If OCTs had been obtained one-month post-diagnosis (when some patients still exhibited papilledema and ongoing congestion) and again after 3–6 months (with no congestion), they might have observed PHOMS disappearance in some patients (45).

In conclusion, it seems more likely that PHOMS serve as a concomitant marker rather than an etiological factor in IIH (15). It may be possible to indirectly assess intracranial pressure levels by monitoring the reduction or disappearance of PHOMS. If non-invasive OCT follow-ups tracking PHOMS changes could determine IIH treatment efficacy, thereby avoiding multiple invasive lumbar punctures in the short term, this would hold significant clinical value. Further investigation is required to elucidate the relationship between visual field defects in IIH patients and PHOMS.

#### 2.4.3. Association between PHOMS and optic neuritis

PHOMS have been reported in inflammatory conditions affecting the optic nerve, with MS being the most prevalent example (46). MS is an autoimmune demyelinating disorder targeting central nervous system, frequently impacting cerebral white matter, spinal cord, brainstem, cerebellum, and optic nerves. Clinical presentations encompass visual impairments, limb weakness, sensory anomalies, and ataxia. This ailment exhibits a heightened prevalence in Europe and North America (29).

In 2020, a prospective longitudinal study of 212 patients with MS and 117 patients without MS found that PHOMS were present prospectively in 10% of eyes affected by optic neuritis and in 0% of 62 healthy controls (10). During a two-year assessment period, while most PHOMS in this study remained unchanged, instances of size enlargement and *de-novo* formation were observed. This finding may suggest that PHOMS can be acquired, grown, and evolve in relation to optic nerve disturbances. Further research is underway to explore the potential association with intermittent intracranial pressure elevation during the MS course and whether optic neuritis relapses correlate with PHOMS enlargement. The study also demonstrated that PHOMS had no impact on optic disc RNFLT and macular GCLT in patients with MS-related optic neuritis. A separate investigation involving MS patients revealed a significant correlation between PHOMS and extended disease duration, as well as a higher Expanded Disability Status Scale in individuals with primary progressive MS, but not in those with early relapsing-remitting MS (29).

The results from the aforementioned studies, together with prior observations of axonal swelling in specific MS patients, suggest that PHOMS could symbolize a delayed manifestation of axonal dysfunction in these individuals, possibly serving as an indicator of disease severity and prognosis.

#### 2.4.4. Association between PHOMS and NA-AION

In the clinical context of NA-AION, PHOMS may serve as a signal underlying axonal dysfunction, offering valuable insights for evaluating disease severity and informing therapeutic decisions. NA-AION is a common, vision-impairing disorder resulting from ischemic injury to the anterior optic nerve. Clinically, NA-AION manifests as sudden, painless vision impairment accompanied by optic disc edema, peripapillary hemorrhages, and, frequently, altitudinal visual field defects (47).

In NA-AION patients, PHOMS manifested in 28% of 36 eyes, independent of concurrent optic nerve pathologies like ODD (6). In 2021, Dai et al. observed a 13% prevalence of PHOMS in central retinal vascular occlusion and a 56% prevalence in NA-AION cases. A significantly greater probability of PHOMS presence was observed in patients with NA-AION compared to those experiencing retinal vascular occlusions. The heightened incidence of NA-AION could be attributed to its frequent manifestation in individuals with crowded, diminutive optic discs, potentially exacerbating axoplasmic stasis following edema and thus increasing the likelihood of PHOMS development (47).

Presently, it is believed that PHOMS may indicate more severe optic disc edema in NA-AION cases, and as the acute phase subsides and edema recedes, PHOMS may noticeably diminish (43). In 2020, Hamann et al. carried out a cross-sectional study involving 65 patients (127 eyes) under 50 years old with NA-AION, identifying a PHOMS prevalence of approximately 28% in NA-AION eyes without ODD and 54% in those with ODD. The presence or absence of PHOMS did not significantly impact retinal nerve fiber layer thickness or ganglion cell layer thickness among these patients. Presently, PHOMS is considered an indicator of more severe optic disc edema in NA-AION cases. As the acute phase subsides and edema recedes, PHOMS may notably diminish. Further research is required to ascertain whether the presence of PHOMS can predict visual prognosis in NA-AION patients during the convalescent phase (6).

#### 2.4.5. Association between PHOMS and TDS/myopia

In a recent comparative study between children with and without PHOMS, a notable relationship was identified between PHOMS and myopia, with optic disc tilt emerging as the unifying factor (7).

Before the term PHOMS was established in 2018, one of the earliest studies describing PHOMS emerged in 2014, focusing on pediatric patients with TDS (4). TDS, an optic disc anomaly linked to myopia, manifests in a modest proportion (1–2%) of the general population (4, 48). Due to their high occurrence, TDS represent a significant cause of pseudopapilledema in clinical settings and, as a result, are frequently associated with the presence of PHOMS (4, 18). In TDS, PHOMS are referred to as dome-shaped reflective structures, with 39.5% (15 eyes) exhibiting this morphological feature on the vertical OCT B-scan (19).

In TDS, nerve fiber damage is particularly susceptible at two sites: the entrance of the scleral canal and the level of the lamina cribrosa. Initially, the Bruch membrane projects toward the optic nerve at its superior extent, forcing the optic nerve fibers to bend sharply as they descend into the scleral canal (4, 49). Subsequently, the nasal displacement of the lamina cribrosa relative to the Bruch's membrane opening (BMO) and the gradual stretching of the sclera and lamina cribrosa—believed to underpin the pathogenesis of optic disc tilt—may induce focal stress on optic nerve axons as they traverse the pores of the lamina cribrosa, particularly in fibers serving the nasal optic disc (4, 50, 51). Therefore, the abnormal morphology predisposes to chronic axoplasmic stasis. To some extent, chronic axoplasmic stasis arises, resulting in the recognizable S-shaped peripapillary bulge of enlarged, vacuolated nerve fibers forming nasally. This leads to nasal pseudopapilledema and optic disc elevation, which are discernible ophthalmoscopically and on end face OCT as a characteristic C-shaped halo. Cross-sectional EDI-OCT through this halo reveals anomalous disk-associated PHOMS (4, 30, 41).

In 2014, a study identified 'dome-shaped hyper-reflective structure(s)' in 39.5% of pediatric patients with TDS (4). Notably, the authors observed that in seven of the 15 eyes, the size of the structure decreased over a 1-year period. Additionally, the study revealed that 46.7% of eyes with visual field abnormalities did not improve after refractive correction, suggesting potential damage resulting from persistent nerve fiber bending and axoplasmic flow impairment. Since then, further studies have investigated the connections between PHOMS, myopia, and tilted discs.

In 2020, Lyu et al. carried out a cross-sectional study that included 66 children with PHOMS and 46 control subjects, investigating the characteristics of children with PHOMS and evaluating associated risk factors (7). This pioneering study identified the degree of myopia, and optic nerve head tilt angle as two notable risk factors for PHOMS. Lyu and colleagues evaluated specific instances at various time points across multiple years, witnessing the emergence of PHOMS in conjunction with myopic shifts during adolescence. This *de-novo* development of PHOMS implies an acquired, rather than a congenital, origin. Lyu and colleagues postulate that myopic shifts in adolescents may be linked to the formation of PHOMS, with optic disc tilt possibly serving as an intermediary between myopia and PHOMS. Optic disc tilting may also result in axonal compression and disrupted axonal transport on the nasal aspect of the optic nerve head, both of which might contribute to the development of PHOMS. This finding was corroborated in a 2022 study by Kim et al., who also documented the *de-novo* emergence of PHOMS during adolescent myopic shifts (52). This case series, monitoring two pediatric patients longitudinally, illustrated the morphological evolution of PHOMS in conjunction with myopic shifts.

Given the evidence supporting the acquired nature of PHOMS, longitudinal investigations in pediatric patients with myopia could prove crucial in elucidating the development of PHOMS, as well as determining whether they are associated with visual outcomes or merely serve as a structural marker of pathology (7, 41, 52). Furthermore, it is essential to explore the potential of quantitative research on PHOMS as a biological marker for myopia progression and investigate whether observing the presence and size of PHOMS in children can predict changes in myopia. In the context of TDS, examining the correlation between the existence or dimensions of nasal PHOMS and temporal visual field defects will provide valuable insights.

### 2.5. Future directions

PHOMS constitute a recent enigmatic discovery within the realm of ophthalmology. These morphological features, discernible on OCT scans, bear considerable implications for various ophthalmic conditions. Despite the burgeoning body of research dedicated to PHOMS, numerous questions persist, warranting further exploration of their clinical relevance, associations with ocular disorders, and prognostic value.

To elucidate the significance of PHOMS and its potential impact on patient care, future investigations should endeavor to address several pivotal avenues of inquiry. Firstly, ascertaining the prevalence of PHOMS in the general population is paramount for determining whether these structures constitute an independent pathology or a sequela of existing ophthalmic conditions. Secondly, examining the correlation between PHOMS size or location and visual field deficits in ODD may yield valuable insights into the pathophysiology of this disorder, as a study has already shown the feasibility of measuring PHOMS volume and its potential significance in correlating with anatomic optic nerve head characteristics for future research (53).

Lastly, with regard to prognostic significance, it is imperative to discern whether PHOMS, as indicators of axoplasmic stasis, can serve as autonomous risk factors for predicting disease outcomes or as harbingers of disease progression. This necessitates the evaluation of PHOMS in relation to vision-compromising conditions such as NA-AION or retinal vascular occlusion, as well as the assessment of their potential to forecast recurrence. Furthermore, determining the capacity of PHOMS to predict papilledema recurrence in IIH and the utility of quantitative PHOMS measurements (surface area and volume) in prognosticating visual outcomes in neuro-ophthalmic disorders is crucial. Finally, it is essential to ascertain whether quantitative assessments of PHOMS can function as a biomarker for myopia progression.

## Author contributions

Conceptualization and drafting of the article: BL. Literature search: HL and QH. Final approval: BL and YZ. All authors contributed to the article and approved the submitted version.
